# A Late Form of Nucleophagy in *Saccharomyces cerevisiae*

**DOI:** 10.1371/journal.pone.0040013

**Published:** 2012-06-29

**Authors:** Dalibor Mijaljica, Mark Prescott, Rodney J. Devenish

**Affiliations:** Department of Biochemistry and Molecular Biology, School of Biomedical Sciences, Faculty of Medicine, Nursing and Health Sciences, Monash University, Victoria, Australia; Texas A&M University, United States of America

## Abstract

Autophagy encompasses several processes by which cytosol and organelles can be delivered to the vacuole/lysosome for breakdown and recycling. We sought to investigate autophagy of the nucleus (nucleophagy) in the yeast *Saccharomyces cerevisiae* by employing genetically encoded fluorescent reporters. The use of such a nuclear reporter, n-Rosella, proved the basis of robust assays based on either following its accumulation (by confocal microscopy), or degradation (by immunoblotting), within the vacuole. We observed the delivery of n-Rosella to the vacuole only after prolonged periods of nitrogen starvation. Dual labeling of cells with Nvj1p-EYFP, a nuclear membrane reporter of piecemeal micronucleophagy of the nucleus (PMN), and the nucleoplasm-targeted NAB35-DsRed.T3 allowed us to detect PMN soon after the commencement of nitrogen starvation whilst delivery to the vacuole of the nucleoplasm reporter was observed only after prolonged periods of nitrogen starvation. This later delivery of nuclear components to the vacuole has been designated LN (late nucleophagy). Only a very few cells showed simultaneous accumulation of both reporters (Nvj1p-EYFP and NAB35-DsRed.T3) in the vacuole. We determined, therefore, that delivery of the two respective nuclear reporters to the vacuole is temporally and spatially separated. Furthermore, our data suggest that LN is mechanistically distinct from PMN because it can occur in *nvj1*Δ and *vac8*Δ cells, and does not require *ATG11*. Nevertheless, a subset of the components of the core macroautophagic machinery is required for LN as it is efficiently inhibited in null mutants of several autophagy-related genes (*ATG*) specifying such components. Moreover, the inhibition of LN in some mutants is accompanied by alterations in nuclear morphology.

## Introduction

Autophagy is an evolutionary conserved, catabolic process responsible for the non-selective or selective degradation of diverse cargoes. In non-selective autophagy bulk cytosol and other cellular components are targeted for degradation. By contrast during selective autophagy, a specific cargo such as a particular organelle is exclusively subjected to autophagic degradation (reviewed in [1]–[4]).

Selective autophagic degradation of the nucleus (nucleophagy) in yeast (*Saccharomyces cerevisiae*) has been categorized as occurring by a microautophagic process, PMN, based on the morphological distinction that the cargo destined for degradation within a nuclear bleb is directly engulfed and sequestered into an invagination of the vacuolar membrane rather than being packaged into autophagosome-like vesicles [5], [6]. Upon nitrogen starvation the initiation of PMN occurs at nucleus-vacuole (NV) junctions formed by interactions between the outer nuclear membrane protein, Nvj1p and the vacuolar membrane protein, Vac8p [5]–[7]. PMN takes place through a series of morphologically distinct steps. First, an NV junction forms at the nuclear envelope (including both inner and outer nuclear membranes), coincident with an invagination of the vacuolar membrane that bulges into the vacuolar lumen. Later a fission event releases into the vacuolar lumen a nuclear-derived vesicle (PMN vesicle) filled with nuclear material enclosed by both nuclear membranes. Eventually, the PMN vesicle is degraded by resident vacuolar hydrolases [5], [7]–[9].

As demonstrated by studies *in vitro*, ‘classical/canonical microautophagy’ (CM) in *S*. *cerevisiae* operates via a specialized structure, the autophagic tube, a long, narrow invagination of the vacuolar membrane that pinches off to form vesicles containing bulk cytosolic material within the lumen of the vacuole. This process is largely independent of the core autophagy-related genes (*ATG*) [10]. By contrast, efficient PMN requires core *ATG* genes [7], [9]. Some additional, non-Atg proteins, such as the components of the vacuolar transporter chaperone (VTC) complex [11], [12] and the exit from rapamycin-induced growth arrest (EGO) complex [13] are reported to be indispensable for CM, but are not required for efficient PMN [7].

In a separate study we have shown that n-Rosella, a pH-based biosensor, can be used in conjunction with fluorescence microscopy to monitor uptake of nucleoplasm into the vacuole [14], [15]. Under growing conditions wild type, BY4741 cells expressing n-Rosella exhibit a pattern of fluorescence consistent with the uniform labelling of the nucleoplasm. In such cells the nucleus appears as a sharply defined single rounded structure that fluoresces both red and green. Nitrogen-starved, wild type cells expressing n-Rosella showed the accumulation of diffuse red fluorescence in the vacuole that indicated the delivery of n-Rosella to the vacuole [14], [15].

Here, we report an extensive analysis of a process that we designate late nucleophagy (LN). Dual labeling of cells with Nvj1p-EYFP, an outer nuclear membrane reporter [5] and NAB35-DsRed.T3, a nucleoplasm reporter has enabled us to demonstrate that induction of PMN can be detected as early as after 3 hours of nitrogen starvation as reported previously [5]. LN can be detected only after prolonged periods of nitrogen starvation (20–24 hours) as observed by both confocal microscopy and immunoblotting. In addition to this clear temporal distinction between the two processes, our data suggest that they are also spatially separated, as we rarely observe labeling of single nuclear-derived, vesicle-like structures located in the vacuole with both reporters. Furthermore, in contrast to PMN, LN can occur in the absence of Nvj1p or Vac8p, and does not require Vps34 PtdIns(3)P-kinase complex I components (Vps34p, Vps15p, Atg6p, and Atg14p) or Atg11p (an autophagy adapter protein). Nevertheless, a subset of the components of the core macroautophagic machinery is shown to be required for LN as it is efficiently inhibited in some *atg* mutants. Interestingly, this inhibition of LN was accompanied by morphological alterations of the nucleus.

## Materials and Methods

### Strains and Plasmids

*S. cerevisiae* strains used in this study were wild type, BY4741 (*MAT* ***a*** *his3*Δ1 *leu2*Δ0 *met15*Δ0 *ura3*Δ0) and the related isogenic single gene deletion strains (Research Genetics™).

Plasmid pAS1NB-NAB35-Rosella (n-Rosella) encodes a dual color-emission pH-biosensor that consists of a pH-stable variant of the red fluorescent protein and pH-sensitive variant of GFP (pHluorin) fused to the C-terminus of NAB35 which encompasses the Nab2p nuclear localization signal [14], [15]. pAS1NB-NAB35-DsRed.T3 encodes only the red fluorescent component of Rosella fused to NAB35 [14], [15]. Plasmid P_CUP1_-Nvj1-EYFP expresses Nvj1p-EYFP under *CUP1* promoter control [5], [16]. Plasmid pGFP-C-FUS expresses cytoplasmic GFP (c-GFP) under *MET25* promoter and *CYC1* terminator control [17].

Plasmid pRS316 was used to express Atg4 or its enzymatically inactive variants (Atg4C159A and Atg4C159S) [18]. pRS416 was used to express the *ATG3* open reading frame under the control of its native promoter and terminator. The *ATG3* gene cassette flanked by 5′ *Hin*dIII and 3′ *Not*I restriction sites was amplified from yeast genomic DNA by PCR using the primer pair ATG3UP (5′- AGAAGCTTACGTTTTCTACCGTTCCCGTCTC) and ATG3DO (5′ATAGTTTAGCGGCCGCTTTACCAACCTTCCATGGTATAG). Following *Hin*dIII/*Bam*HI digestion the PCR product was ligated into the expression site of pRS416 [19].

Transformation of yeast cells with plasmid DNA was performed as described previously [15].

### Media and Growth Conditions

For the purpose of fluorescence imaging yeast strains were grown as previously described [14], [15]. Briefly, cell cultures were grown to mid-exponential growth phase in Saccharomyces Salts medium (SS) with the addition of 2% (w/v) glucose (SS+D) as a carbon source. Cells were harvested and washed three times with SS+D medium and either re-inoculated into fresh SS+D medium, or nitrogen starvation medium (SD-N) containing 0.17% (w/w) yeast nitrogen base without added amino acids or ammonium sulfate and containing 2% (w/v) glucose. Amino acid supplements were added as required.

Basal levels of Nvj1p-EYFP expression under *CUP1* promoter control were achieved by growth in SS+D without added Cu^2+^. Expression was induced by growth in medium containing 0.1 mM CuSO_4_ for 1 hour as previously described [5], [16].

### Fluorescence Microscopy

Cells were prepared as described previously [14], [15]. To label the vacuole lumen, cells were stained by incubation with CMAC-Arg dye (Invitrogen-Molecular Probes, Cat. No. Y-7531) as described previously [14], [15]. Nuclei were stained by incubation with the nucleic acid stain Hoechst 33258 (Invitrogen-Molecular Probes, Cat. No. H1398) at a final concentration of 1 µM for 45 min immediately before imaging. Confocal Laser Scanning Microscopy (CLSM) was performed on a Fluoview FV500 microscope (Olympus, Australia) using published parameters [14], [15]. Yeast cells expressing either n-Rosella or NAB35-DsRed.T3 were scored for the delivery of diffuse red fluorescence into the vacuole concomitant with checking for the absence of green fluorescence as appropriate. At the same time these cells were scored for nuclear morphology (normal  =  round nucleus; altered  =  irregularly shaped nucleus). Yeast cells expressing Nvj1p-EYFP were scored for the accumulation in the vacuole of blebs and vesicle-like structures showing yellow fluorescence emission (EYFP is considered yellow, but it is detected in the ‘green’ channel). In each experiment 200–300 cells were scored and experiments were performed in triplicate.

### Immunoblotting

Cells expressing n-Rosella (OD_600_ = 2.5) were harvested by centrifugation and resuspended in 100 µl water. NaOH (100 µl, 0.2 M) was added and the cell suspension incubated for 5 min at 21°C and centrifuged. The pellet was resuspended in 50 µl SDS-PAGE sample buffer (0.06 M Tris-HCl, pH 6.8, 5% (v/v) glycerol, 2% (w/v) SDS, 4% (v/v) β-mercaptoethanol, 0.0025% (w/v) bromophenol blue), boiled for 3 min and centrifuged when cool. Sample supernatants (5 μl) were subjected to SDS-PAGE on 12% polyacrylamide gels. Proteins were transferred to PVDF membranes and probed with a monoclonal antibody against GFP (1∶4,000 dilution) (Roche Applied Science, Cat. No. 11814460001), or 3-phosphoglycerate kinase (1∶2,000 dilution) (Molecular Probes-Invitrogen, Cat. No. A6457). Secondary antibody was HRP-conjugated anti-mouse IgG (GE Healthcare, Cat. No. NA931V) (1∶20,000 dilution). Chemiluminescent signals were generated using ECL reagent (ThermoScientific, Cat. No. 34095) and detected using photographic film.

After exposure to ECL, the membranes were washed in 10% (v/v) methanol, for 30 min at room temperature. Then, additional washing of the membranes was performed for 30 min (2×15 min) with wash buffer (1×PBS). A stripping procedure was performed for 30 min by washing in pre-warmed (37°C) stripping buffer, pH 2.2 (containing 1.5% (w/v) glycine, 0.1% (w/v) SDS and 1% (v/v) Tween-20) for 30 min. Then the membranes were washed for 30 min (3×10 min) with wash buffer (1×PBS), then blocked for 30 min in blocking buffer (1×PBS containing 5% (w/v) skim milk powder). In all experiments we first carried out blotting for GFP, stripped the membranes as described above and then blotted for PGK (3-phosphoglycerate kinase).

### Electron Microscopy

For the purpose of electron microscopy cells were grown aerobically at 28°C to mid-exponential growth phase in SS+D medium containing the required auxotrophic supplements. Cells were harvested and washed three times with SS+D medium and re-inoculated into fresh SS+D or SD-N medium and incubated at 28°C for the times indicated.

Sample preparation procedures including fixing, embedding and ultrathin sectioning with lead citrate staining were according to Bauer *et al.* [20]. Samples were visualized on a Jeol 101 TEM electron microscope (Japan). Images were processed and analyzed using Image J (version 1.36b) (http://rsb.info.nih.gov/ij/).

## Results

### Delivery of n-Rosella to the Vacuole is Only Apparent after Prolonged Periods of Nitrogen Starvation

**Figure 1:**
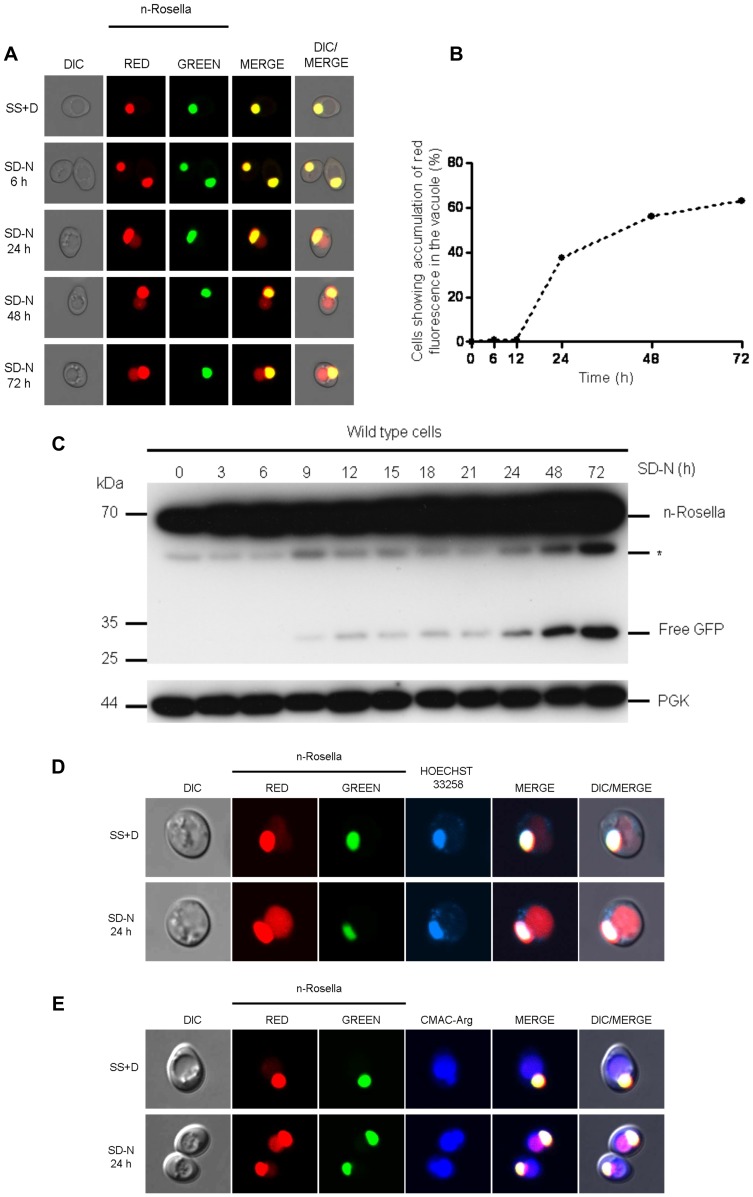
Delivery of n-Rosella to the vacuole of nitrogen-starved wild type cells. (**A**) Wild type (BY4741) cells expressing n-Rosella were subjected to nitrogen starvation and then sampled at selected time points (0, 6, 24, 48 and 72 hours after commencement of nitrogen starvation). Accumulation of diffuse red fluorescence in the vacuole was taken as evidence of nucleophagy. (**B**) The percentage of cells showing accumulation of diffuse red fluorescence in the vacuole at various time points is shown. (**C**) Cells expressing n-Rosella were starved in SD(-N) medium for 0, 3, 6, 9, 12, 15, 18, 21, 24, 48 and 72 hours, and the increase in processed degradation product (free GFP) levels was monitored by immunoblotting as described in Materials and Methods. Cytosolic PGK was detected as a loading control. *indicates the presumptive degradation product of n-Rosella lacking the NAB35 nuclear targeting signal. (**D**) Staining with Hoechst 33258 was performed to confirm the targeting of n-Rosella (red and green fluorescence) to the nucleus 24 hours after commencement of nitrogen starvation. (**E**) Staining with the vacuolar lumen dye, CMAC-Arg was performed to confirm the delivery of n-Rosella (diffuse red fluorescence) to the vacuole (24 hours after commencement of nitrogen starvation).

Nucleophagy was induced in wild type cells by incubating in SD-N medium. Cells were sampled at selected time points between 0 and 72 hours after the onset of nitrogen starvation and examined using fluorescence microscopy. The accumulation of n-Rosella (diffuse red fluorescence) in the vacuole was considered to be indicative of the occurrence of nucleophagy (Fig. 1A–E) [14]. The delivery of n-Rosella to the vacuole could only be observed after prolonged periods of nitrogen starvation (Fig. 1A–C). After 24 hours ∼42% of cells showed accumulation of diffuse red fluorescence in the vacuole (Fig. 1B), while prolonged periods of nitrogen starvation (48 and 72 hours) lead to a higher proportion of cells showing evidence of nucleophagy (56% and 63%, respectively) (Fig. 1B). The correct targeting of n-Rosella (red and green fluorescence) to the nucleus was confirmed by co-localization with blue fluorescence emission of Hoechst 33258 used to label nucleic acids (Fig. 1D). The accumulation of diffuse red fluorescence was confirmed as being localized inside the vacuole on the basis of co-localization with the vacuole lumen marker, CMAC-Arg (Fig. 1E).

Furthermore, we followed the proteolytic digestion of n-Rosella by immunoblotting using an antibody with specificity for the GFP component. n-Rosella is comprised of a nuclear targeting signal (∼ 5 kDa), two fluorescent proteins (each ∼28 kDa) linked by a short exposed polypeptide (∼1 kDa). Delivery of n-Rosella to the vacuole exposes it to the lumenal proteases resulting in several cleavage products. Cleavage of the polypeptide linker that joins the two fluorescent protein moieties will lead to the production of ‘free’ GFP which serves as a measure of vacuolar degradation of n-Rosella. Cell lysates prepared from cells harvested at selected time points between 0 and 72 hours after the onset of nitrogen starvation were subjected to SDS-PAGE and after transfer of proteins to PVDF membranes blots probed for GFP (Fig. 1C). The predominate band observed for both non-starved and starved cells migrated as a polypeptide ∼ 65 kDa and is presumed to be full-length n-Rosella. A band denoted * migrates with a size corresponding to ∼58 kDa which is consistent with it being n-Rosella lacking the NAB35 nuclear targeting signal (Fig. 1C). At time points between 9 and 21 hours of nitrogen starvation, small amounts of a band migrating with a size corresponding to ∼28 kDa, consistent with the proteolytic release of free GFP were observed. The amount of GFP released by digestion of n-Rosella was significantly increased at 24 hours of starvation and later time points (Fig. 1C). Collectively, these results are consistent with delivery of n-Rosella to the vacuole. Cytosolic PGK, migrating with a size corresponding to ∼44 kDa, served as a loading control.

We next studied wild type cells of two further strains having different genetic backgrounds, YRD15 [21] and CRY1 [22]. The delivery of n-Rosella to the vacuole of both YRD15 and CRY1 cells as judged by accumulation of vacuolar red fluorescence was observed only after 20–24 hours following commencement of nitrogen starvation. Furthermore, the proportion of YRD15 and CRY1 cells showing evidence of nucleophagy was 20 and 25–35%, respectively (data not shown), comparable to that for BY4741 cells. These results suggest that the late onset of nucleophagy revealed using n-Rosella is independent of strain background.

### Evidence for Direct Delivery of NAB35 Targeted Reporters to the Vacuolar Lumen

**Figure 2:**
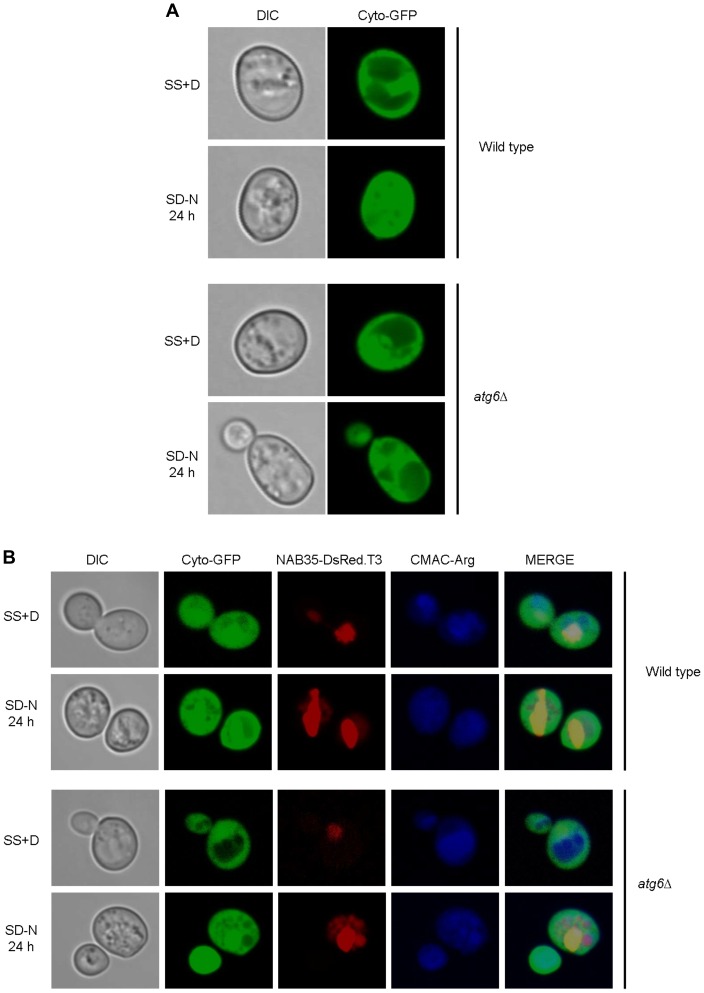
The diffuse red fluorescence (NAB35-DsRed.T3) accumulated in the vacuole is delivered directly from the nucleus. Wild type (BY4741) and *atg6*Δ cells expressing cyto-GFP alone (**A**), or co-expressing cyto-GFP and NAB35-DsRed.T3 (**B**), under growing (SS+D) and nitrogen starvation conditions (SD-N) were sampled after 24 hours. Staining with the vacuolar lumen staining dye, CMAC-Arg was used to confirm delivery of fluorescent reporters to the vacuole.

We sought to exclude the possibility that NAB35 targeted reporter proteins either ‘leak’ out of the nucleus, or are mis-targeted to the cytoplasm for some unknown reason after the prolonged incubation of the cells in nitrogen starvation medium, and are then transported to the vacuole via a canonical autophagic pathway. To this end we conducted experiments using *atg6*Δ cells. Atg6 is regarded as a core component of the autophagy machinery and as such both macroautophagy and microautophagy would be non-functional in these cells [23]. This phenotype was confirmed by expression in *atg6*Δ cells of GFP localized in the cytoplasm. Such cells did not show green fluorescence in the vacuole after 20 (or more) hours of nitrogen starvation (Fig. 2A). As indicated *atg6*Δ cells are capable of LN, but not PMN. Dual-labeled cells expressing both cytoplasmically localized GFP and nuclear localized NAB35-DsRed.T3 (the red fluorescent component of n-Rosella biosensor) grown under the same conditions of nitrogen starvation showed red fluorescence in the vacuole, but not green fluorescence (Fig. 2B). Therefore, we conclude that the red fluorescent material accumulated in the vacuole is n-Rosella delivered directly from the nucleus.

### LN and PMN are Initiated at Different Times During Nitrogen Starvation

In wild type cells PMN was observed to occur 3 hours after the commencement of nitrogen starvation [5], [7]. The relatively late time point (24 hours and beyond) at which delivery of n-Rosella to the vacuole of nitrogen-starved wild type cells could be detected suggested the LN process we were observing was different to that of PMN. Thus, we next sought to determine if it was indeed the case that the timing of LN is distinctly separate from the timing of PMN in starved wild type cells. Cells harboring vectors encoding NAB35-DsRed.T3 and Nvj1p-EYFP (under control of a Cu^2+^ inducible promoter) individually, or together, were grown in SS+D medium. Expression of Nvj1p-EYFP was induced for 1 hour by the addition of CuSO_4_ to the growth medium before transfer to SD-N medium. Control cells were incubated in fresh SS+D lacking exogenous Cu^2+^ for the same time period.

**Figure 3:**
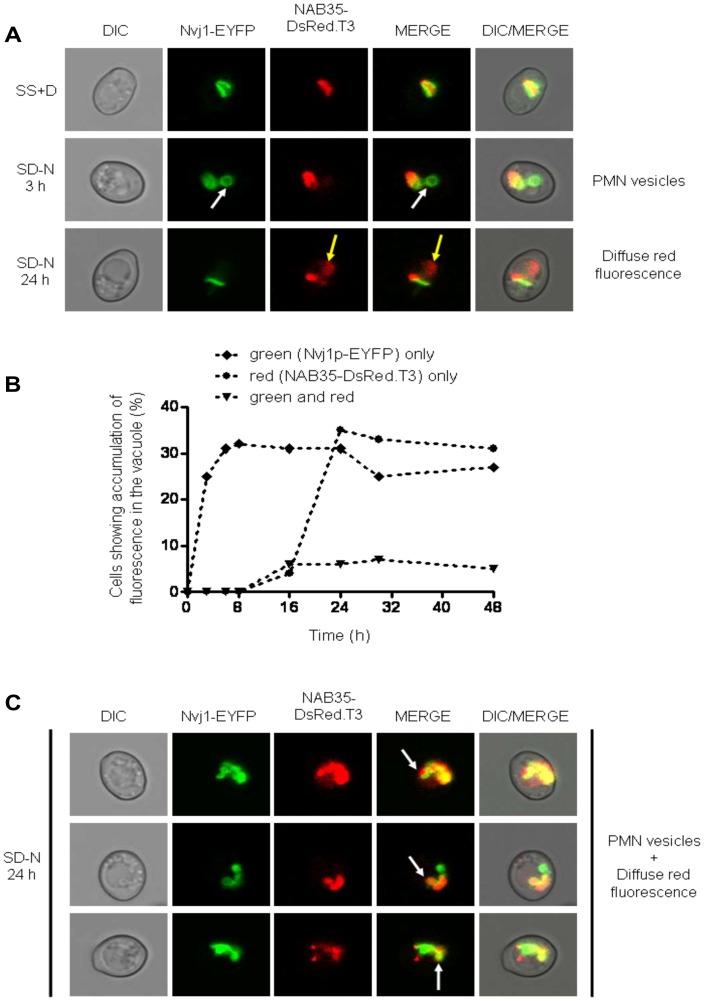
Wild type cells co-expressing Nvj1-EYFP and NAB35-DsRed.T3 nuclear reporters show temporal separation of nucleophagic events. (**A**) Wild type (BY4741) cells co-expressing both nuclear reporters were imaged under growing (SS+D) and nitrogen starvation (SD-N) conditions (3 and 24 hours after commencement of nitrogen starvation), respectively. The appearance of Nvj1p-EYFP-derived vesicles (PMN vesicles) in the vacuole is highlighted by white arrows, whereas accumulation of NAB35-DsRed.T3-derived fluorescence (diffuse red fluorescence) is indicated by yellow arrows. (**B**) Percentage of cells showing accumulation of fluorescence in the vacuole over time: ♦ green (Nvj1p-EYFP) only; • red (NAB35-DsRed.T3) only; ▾ green and red. (**C**) Accumulation of both Nvj1p-EYFP-derived vesicles (PMN vesicles) and accumulation of NAB35-DsRed.T3-derived diffuse red fluorescence in the same cells 24 hours after commencement of nitrogen starvation. The appearance of vacuolar vesicles containing both nuclear reporters is indicated by white arrows.

When both the reporters NAB35-DsRed.T3 and Nvj1p-EYFP were expressed together in wild type cells, Nvj1p-EYFP labeled PMN vesicles (Fig. 3A; white arrows and Fig. S1A) could be detected in the vacuole as early as after 3 hours of nitrogen starvation, whereas delivery of NAB35-DsRed.T3 could be detected as diffuse red fluorescence only after 20–24 hours (Fig. 3A; yellow arrows). A time course of detection of both events is presented in Fig. 3B. Notably, vesicles labeled with both Nvj1p-EYFP and NAB35-DsRed.T3 were not observed in cells subjected to starvation for 20 hours or less. However, co-labeling of one or two vesicles in the vacuole was observed in a small percentage (∼5%) of cells from 20 hours and beyond (Fig. 3C; white arrows and Fig. S1B). These results indicate that the delivery of each reporter is initiated at different time points after the onset of nitrogen starvation. The same behavior of reporters has been observed in rapamycin-treated cells (data not shown), indicating that LN is not a process specifically related to growth in nitrogen starvation medium.

Cells expressing Nvj1p-EYFP alone confirmed our observations with dual-labeled cells that PMN can be first detected as early as after 3 hours of the commencement of nitrogen starvation. At this time, as shown previously by Goldfarb and colleagues [5] some 50–60% of cells showed Nvj1p-EYFP-labeled vesicles in the vacuolar lumen. The percentage of cells showing this phenotype remained at this level up to 48 hours following initiation of nitrogen starvation (data not shown). In parallel experiments using cells expressing only NAB35-DsRed.T3 the accumulation of diffuse red fluorescence in the vacuole was observed after 20–24 hours of starvation in 25–35% of cells, as observed for both dual-labeled cells and cells expressing n-Rosella alone (data not shown).

### Delivery of Nucleoplasm to the Vacuole does not Require Nvj1p, Vac8p, or Vac8p-Binding Partners

**Figure 4:**
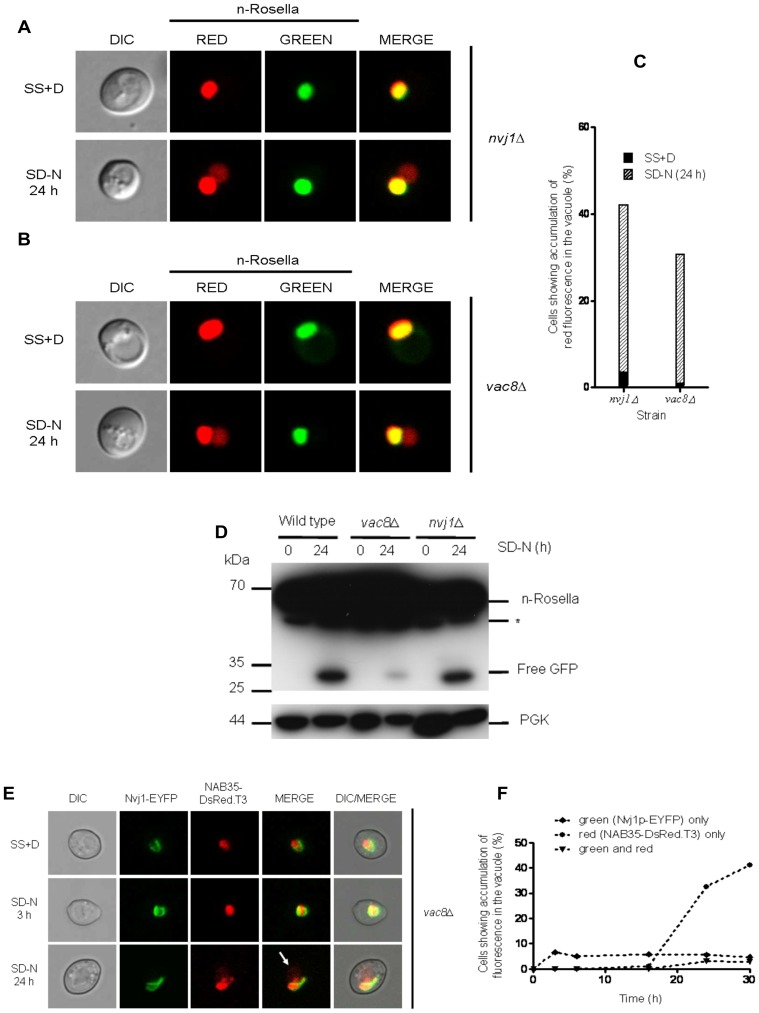
The inactivation of the *NVJ1* and *VAC8* genes required for PMN does not abrogate the delivery of n-Rosella to the vacuole. *nvj1*Δ (**A**) and *vac8*Δ (**B**) cells expressing n-Rosella were imaged under growing (SS+D) and nitrogen starvation (SD−N) conditions (24 hours after commencement of nitrogen starvation). (**C**) The percentage of *nvj1*Δ and *vac8*Δ cells showing accumulation of diffuse red fluorescence in the vacuole under growing (SS+D) and nitrogen starvation (SD-N) conditions (24 hours after commencement of nitrogen starvation). (**D**) Wild type, *nvj1*Δ and *vac8*Δ cells expressing n-Rosella were starved in SD(−N) medium for 0 and 24 hours, and the levels of the free GFP degradation product monitored by immunoblotting as described in Materials and Methods. Cytosolic PGK was detected as a loading control. *indicates the presumptive degradation product of n-Rosella lacking the NAB35 nuclear targeting signal. (**E**) *vac8*Δ cells co-expressing the two nuclear reporters, Nvj1-EYFP and NAB35-DsRed.T3 were imaged under growing (SS+D) and nitrogen starvation (SD−N) conditions (3 and 24 hours after commencement of nitrogen starvation), respectively. The accumulation of NAB35-DsRed.T3-derived fluorescence (diffuse red fluorescence) is indicated by white arrow. (**F**) Percentage of *vac8*Δ cells showing accumulation of fluorescence in the vacuole over time: ♦ green (Nvj1p-EYFP) only; • red (NAB35-DsRed.T3) only; ▾ green and red.

Nvj1p and Vac8p are two proteins found at NV junctions and that are required for mediating PMN [5], [7]. Under conditions of nitrogen starvation *nvj1*Δ (Fig. 4A) and *vac8*Δ (Fig. 4B) cells expressing n-Rosella showed significant accumulation of diffuse red fluorescence in the vacuole after 24 hours of nitrogen starvation (Fig. 4C). Immunoblotting analysis of lysates prepared from *nvj1*Δ or *vac8*Δ cells indicated that free GFP was present in cells only after 24 hours of nitrogen starvation (Fig. 4D). Collectively, these results indicate that LN is not dependent on either the *NVJ1* or *VAC8* gene product.

To corroborate the results obtained using n-Rosella, NAB35-DsRed.T3 and Nvj1-EYFP were expressed individually or co-expressed in *vac8*Δ cells. Upon nitrogen starvation, the delivery of Nvj1p-EYFP to the vacuole was abrogated (and remained so), whereas the delivery of NAB35-DsRed.T3 could still be observed after 24 hours of nitrogen starvation, as expected (Fig. 4E; white arrow and Fig. 4F).

A number of Vac8p-binding partners, identified by yeast two-hybrid screening, are encoded by the *VAC17*, *TCO89*, *ATG13*, *VID21*, *VAB2* and *TAO3* genes, plus two uncharacterized open reading frames, *YEL043W* and *YFR035C* [24]. We investigated whether LN is dependent on these proteins by nitrogen starvation of strains harboring n-Rosella and each lacking expression of a putative Vac8p binding partner. The haploid *tao3*Δ strain is inviable [24] and was not investigated here. *ATG13* encodes a protein considered a component of the core macroautophagic machinery [25], [26]. Delivery of n-Rosella to the vacuole was observed in only a small proportion (∼5%) of *atg13*Δ cells subjected to nitrogen starvation (Fig. S2A); the majority of cells did not accumulate diffuse red fluorescence in the vacuole. Moreover many cells (∼35%) exhibited altered nuclear morphology (see below). Null mutants for the remaining six genes were assessed for their ability to deliver n-Rosella to the vacuole. In each case the outcome, in terms of accumulation of diffuse red fluorescence in the vacuole, was essentially equivalent to that found for wild type cells subjected to nitrogen starvation (Fig. S2B). Lysates of *vac17*Δ and *tco89*Δ cells were subjected to immunoblotting analysis and the results showed that after 24 hours of nitrogen starvation free GFP could be detected (Figs. S2A–B and Fig. S3A). These results indicate that aside from Atg13p the absence of individual putative Vac8p binding partners did not inhibit LN, or lead to altered nuclear morphology (Table 1).

**Table 1:** The requirement for different *ATG* and non-*ATG* genes on the degradation of the nucleus and nuclear morphology during LN.

Gene(s)	Gene group	Requirement for the deliveryof n-Rosella to the vacuole	Alterations in nuclearmorphology
*ATG6*, *ATG11*, *ATG14*, *ATG15* a, *ATG19*, *ATG20*,*ATG21*, *ATG22* b, *ATG24*, *ATG26*, *ATG27*, *ATG32*,*ATG33*, *ATG34*	I	NO	NO
*ATG1* *, *ATG2*, *ATG3*, *ATG4*, *ATG5*, *ATG7* *,*ATG8* *, *ATG9*, *ATG10* *, *ATG12*, *ATG13* *,*ATG16* *, *ATG17* *, *ATG18*, *ATG23* *, *ATG29* *,*ATG31* *	II	YES	YES
*NVJ1*, *VAC8*	PMN	NO	NO
*VAC17*, *TCO89*, *VID21*, *VAB2*, *YEL043W*,*YFR035C*	Vac8 binding partners	NO	NO
*VTC1*, *VTC2*, *VTC3*, *VTC4*, *EGO1*, *EGO3*	Classical Microautophagy	NO	NO
*VPS15*, *VPS34*	Phosphatidylinositol 3-kinase(PI3K) complex I	NO	NO
*YGR223C*	Atg18p-family member	YES/NO	YES/NO

a This mutant shows accumulation of vesicles rather than diffuse red fluorescence in the vacuole.

b *ATG22* is vacuolar amino acid permease required for efflux after autophagic breakdown [29]. This mutant shows accumulation of vesicles and diffuse red fluorescence in the vacuole.

* These mutants show a leaky phenotype in ∼5% of cells (see text for details).

### LN Puncta can be Visualized in the Vacuoles of *atg15*Δ and *pep4*Δ Cells

We next sought evidence that the fluorescent n-Rosella might enter the vacuole in the form of vesicles or particulate structures that are very rapidly broken down such that they are not seen under conditions where the vacuolar enzymes show normal activity. Thus, we investigated the delivery to the vacuole of n-Rosella or both Nvj1p-EYFP and NAB35-DsRed.T3 in *atg15*Δ cells. The *ATG15* gene product is a putative lipase [27], [28] that promotes the lysis of intravacuolar autophagic bodies, cytoplasm-to-vacuole targeting (Cvt) vesicles and multivesicular bodies delivered to the vacuole under autophagy-inducing conditions. Our expectation was that lysis of vesicular or particulate structures would be delayed in *atg15*Δ cells much like it is in *pep4*Δ cells [28].

**Figure 5:**
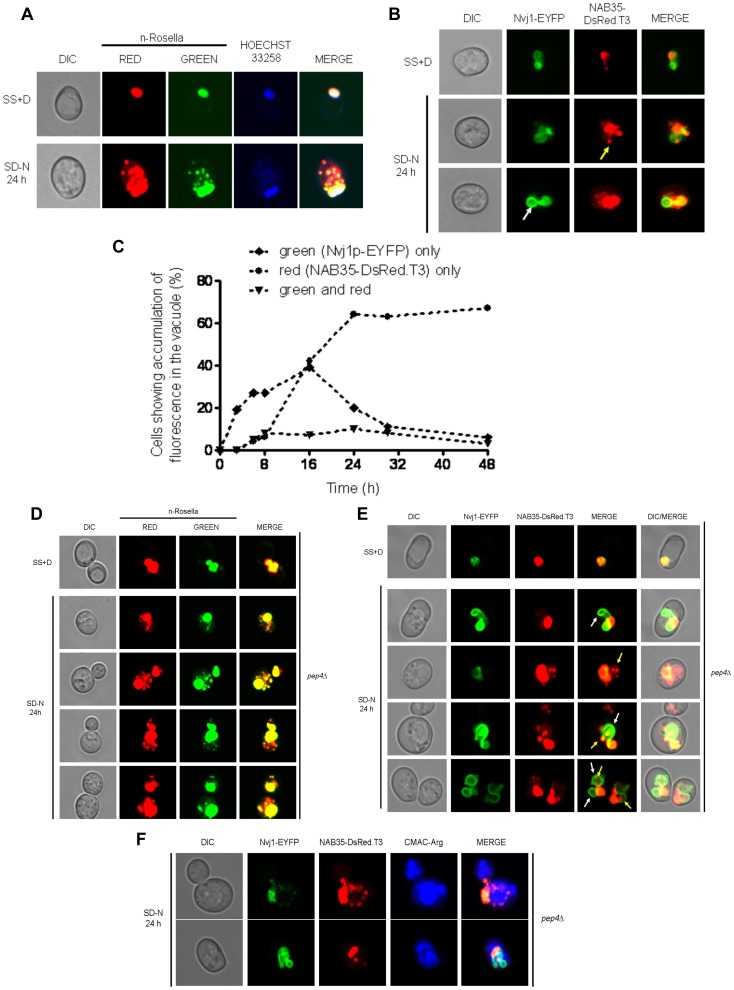
Detection of nuclear-derived intra-vacuolar puncta in *atg15*Δ and *pep4*Δ cells. (**A**) *atg15*Δ cells expressing n-Rosella were imaged under growing (SS+D) and nitrogen starvation (SD−N) conditions (24 hours after commencement of nitrogen starvation). Staining with Hoechst 33258 was performed to confirm the targeting of n-Rosella (red and green fluorescence) to the nucleus and nucleus-derived vesicles/puncta observed in the vacuole. (**B**) *atg15*Δ cells co-expressing the nuclear reporters, Nvj1-EYFP and NAB35-DsRed.T3 were imaged under growing (SS+D) and nitrogen starvation (SD−N) conditions (24 hours after commencement of nitrogen starvation). White arrow highlights Nvj1p-EYFP labeled vesicle whereas yellow arrow highlights NAB35-DsRed.T3 labeled vesicle/puncta, respectively. (**C**) Percentage of cells showing accumulation of reporter fluorescence over time: ♦ green (Nvj1p-EYFP) only; • red (NAB35-DsRed.T3) only; ▾ green and red. (**D**) *pep4*Δ cells expressing n-Rosella were imaged under growing (SS+D) and nitrogen starvation (SD−N) conditions (24 hours after commencement of nitrogen starvation). (**E**) *pep4*Δ cells co-expressing the nuclear reporters, Nvj1-EYFP and NAB35-DsRed.T3 were imaged under growing (SS+D) and nitrogen starvation (SD−N) conditions (24 hours after commencement of nitrogen starvation). The appearance of Nvj1p-EYFP-derived vesicles (PMN blebs and/or vesicles) in the vacuole is highlighted by white arrows, whereas accumulation of NAB35-DsRed.T3-derived vesicles/puncta is indicated by yellow arrows. (**F**) Staining with the vacuolar lumen dye, CMAC-Arg confirmed the delivery of NAB35-DsRed.T3 (red fluorescence) and Nvj1-EYFP (green fluorescence) to the vacuole.

Nitrogen-starved, *atg15*Δ cells expressing n-Rosella exhibited the accumulation of red and/or red/green puncta in their vacuoles after 16 hours of nitrogen starvation (Fig. 5A and Fig. S4A). These puncta were observed to co-localize with Hoechst 33258 nucleic acid stain, indicating that they most likely have derived from the nucleus. The nature of the Hoechst 33258 stained material has not been determined. The observation that some puncta (5–10%) exhibited both red and green fluorescence suggests that their delivery occurs via some form of vesicle-like structure. Presumably their exposure to the acidic conditions of the vacuolar lumen is delayed in the absence of Atg15p thereby allowing the green fluorescence to persist.

To further investigate the origin of these vacuolar puncta, we next investigated nitrogen-starved *atg15*Δ cells expressing both Nvj1p-EYFP and NAB35-DsRed.T3. Nvj1p-EYFP vesicles were found to accumulate in the vacuole 3 hours after onset of starvation, whereas intensely red fluorescent (NAB35-DsRed.T3) puncta were first visualized after 16 hours, but were more evident at 20 hours and beyond. After 24 hours of nitrogen starvation vesicles positive for Nvj1p-EYFP (Fig. 5B; white arrow and Fig. S4B), or NAB35-DsRed.T3 (Fig. 5B; yellow arrow and Fig. S4B), were observed. Notably, only a few cells (<5%) exhibited vesicle-like structures showing both green and red fluorescence indicating the presence of both reporters (Fig. 5C). Collectively, these results suggest that the formation of PMN vesicles and LN vesicle-like structures, are mechanistically distinct. Furthermore, the observation that red puncta are first observed in *atg15*Δ at the same time as diffuse red fluorescence is observed in wild type cells argues against the temporal difference in detection of LN compared to PMN arising simply from differential sensitivity of detection between EYFP-labeled membrane vesicles (PMN) and red fluorescence (LN). We also carried out similar experiments in *pep4*Δ cells in which degradation of material delivered to the vacuolar is also perturbed. The results obtained for *pep4*Δ cells (Fig. 5D–F and Fig. S4C–E) mirror those obtained for *atg15*Δ cells. It is important to note that intravacuolar Nvj1-EYFP labeled vesicles and NAB35-DsRed.T3 derived puncta co-localize with the vacuolar lumen marker CMAC-Arg (Fig. 5F). Immunoblotting analysis of lysates prepared from *atg15*Δ or *pep4*Δ cells did not detect any free GFP even after 24 hours of nitrogen starvation. The absence of degradation product on immunoblots of lysates lacking putative lipase (*atg15*Δ) or vacuolar proteinase A (*pep4*Δ) confirms the vacuolar origin of the GFP degradation product (Fig. S4E).

### Efficient Delivery of n-Rosella to the Vacuole Requires a Spectrum of Core *ATG* Genes

Efficient PMN requires many *ATG* genes [7], [9]. We assessed null mutants of all 31 *ATG* genes known to be involved in autophagic processes in *Saccharomyces cerevisiae*, for LN under conditions of nitrogen starvation (Fig. S2A). In some cases altered nuclear morphology was observed (see below). Based on our observations, we grouped the *ATG* genes into two classes (I and II) depending on whether their deletion affected either their ability to deliver n-Rosella to the vacuole and/or nuclear morphology during nitrogen starvation (Table 1).

**Figure 6:**
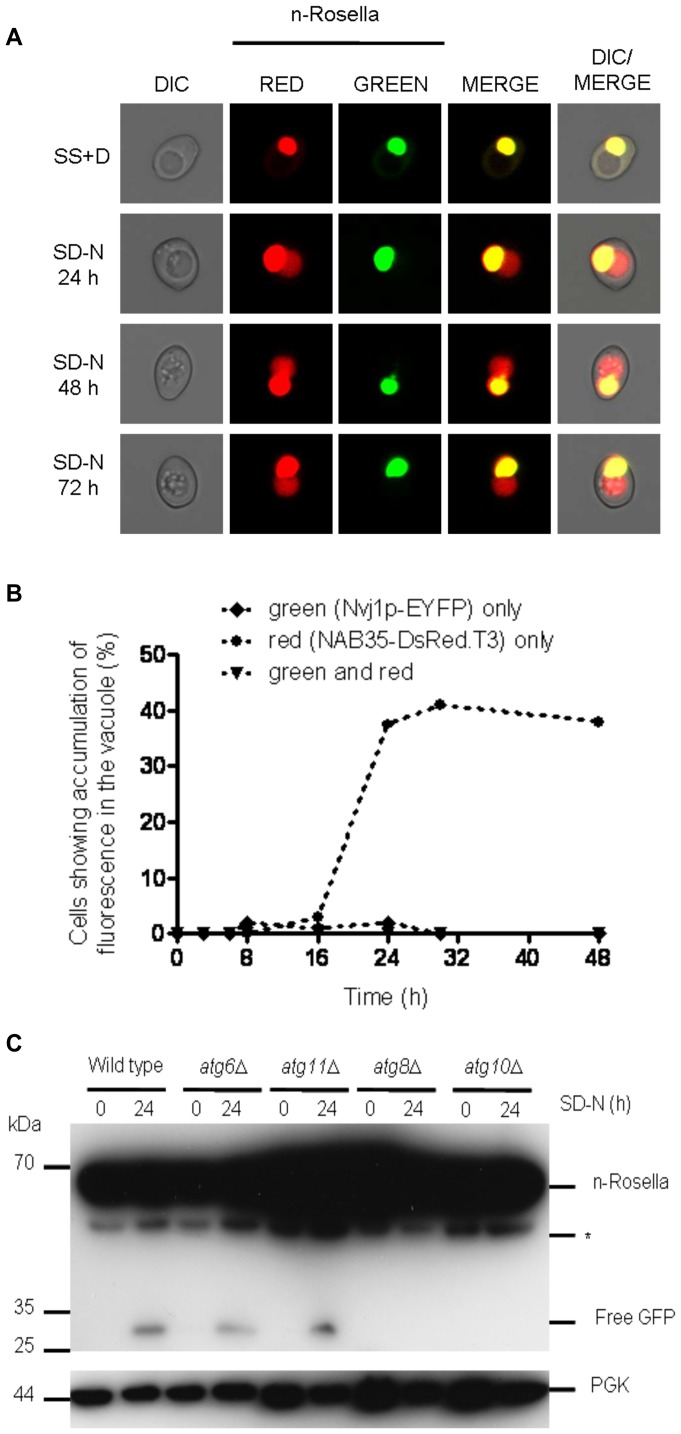
The deletion of the *ATG6* or *ATG11* genes does not abrogate the delivery of n-Rosella to the vacuole. (**A**) *atg11*Δ cells expressing n-Rosella were imaged under growing (SS+D) and nitrogen starvation (SD-N) conditions (0, 24, 48 and 72 hours after commencement of nitrogen starvation). (**B**) *atg11*Δ cells co-expressing both nuclear reporters were imaged under growing (SS+D) and nitrogen starvation (SD-N) conditions and the percentage of cells showing accumulation of fluorescence over time determined: ♦ green (Nvj1p-EYFP) only; • red (NAB35-DsRed.T3) only; ▾ green and red. (**C**) Wild type, *atg6*Δ, *atg11*Δ, *atg8*Δ and *atg10*Δ cells expressing n-Rosella were starved in SD(-N) medium for 0 and 24 hours, and degradation product (free GFP) levels were monitored by immunoblotting as described in Materials and Methods. Cytosolic PGK was detected as a loading control. *indicates the presumptive degradation product of n-Rosella lacking the NAB35 nuclear targeting signal.

Class I genes include: *ATG6*, *ATG11*, *ATG14*, *ATG15*, *ATG19*, *ATG20*, *ATG21*, ATG22* [29], *ATG24*, *ATG26*, *ATG27*, *ATG32*, *ATG33* and *ATG34* (Table 1). Mutant strains exhibit delivery of n-Rosella to the vacuole during nitrogen starvation at levels comparable to the wild type cells (Fig. S2A) and exhibit normal nuclear morphology. Since *ATG11* has been reported to be required for efficient PMN [3], we monitored the delivery of Nvj1p-EYFP and NAB35-DsRed.T3 to the vacuole in *atg11*Δ cells. We confirmed previous observations [7] that PMN is abrogated in *atg11*Δ cells (data not shown). By contrast, the accumulation in the vacuole of n-Rosella (Fig. 6A) and NAB35-DsRed.T3 could still be observed in some 25–35% of cells (Fig. 6B) after 24 hours, as in wild type cells. Immunoblotting analysis of lysates prepared from *atg6*Δ or *atg11*Δ cells detected free GFP only after 24 hours of nitrogen starvation (Fig. 6C and Figs. S2B and S3B). Collectively, these results indicate that functional *ATG* gene products from Class I, including *ATG6* and *ATG11*, are not required for efficient LN.

Class II genes include ATG1*, *ATG2*, *ATG3*, *ATG4*, *ATG5*, ATG7*, ATG8*, *ATG9*, ATG10*, ATG12, ATG13*, ATG16*, ATG17*, ATG18, ATG23*, ATG29* and ATG31*. In null mutants for some of these genes (designated by *), n-Rosella was delivered to the vacuole in a small proportion (∼5–10%) of nitrogen-starved cells that still exhibited normal nuclear morphology [14]. However, the majority of cells for these null mutants for all cells for the other mutants in class II did not accumulate diffuse red fluorescence in the vacuole (Fig. S2A), and ∼35% of nitrogen-starved cells exhibited altered nuclear morphology (Table 1). Instead of containing a round-shaped nucleus such cells exhibited an irregularly shaped nucleus often with projecting ‘arms or horns’. In order to establish if nitrogen starvation affects the changes in nuclear morphology, *atg8*Δ or *atg10*Δ cells expressing n-Rosella were nitrogen starved for 24 hours and then resuspended in SS+D medium (i.e., growth medium) for 6 or 24 hours. At both time points all cells exhibited normal round-shaped nuclear morphology (data not shown). These results indicate that the alteration in nuclear morphology is a consequence of nitrogen starvation in *atg8*Δ [14] and *atg10*Δ cells. Immunoblotting analysis of lysates prepared from *atg8*Δ or *atg10*Δ cells did not detect any free GFP even after 24 hours of nitrogen starvation (Fig. 6C and Figs. S2B and S3C). Together, these results indicate that functional *ATG* gene products from Class II including *ATG8* and *ATG10* are essential for efficient LN.

**Figure 7:**
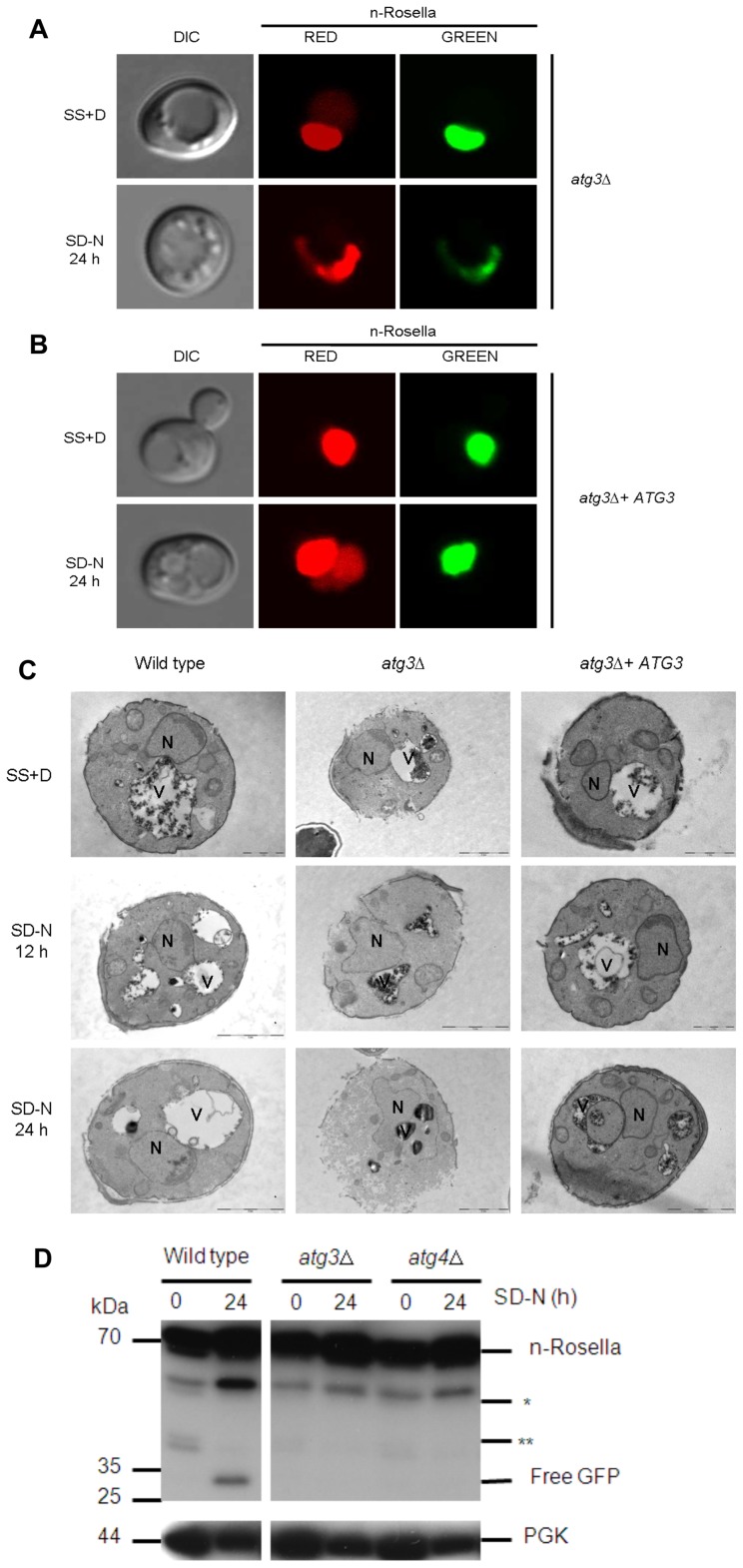
Deletion of the *ATG3* gene abrogates the delivery of n-Rosella to the vacuole and influences nuclear morphology. (**A**) *atg3*Δ and (**B**) *atg3*Δ + *ATG3* cells expressing n-Rosella were imaged under growing (SS+D) and nitrogen starvation (SD−N) conditions (24 hours after commencement of nitrogen starvation). (**C**) Electron microscopy images of wild type (BY4741), *atg3*Δ and *atg3*Δ + *ATG3* cells expressing n-Rosella were imaged under growing (SS+D) and nitrogen starvation (SD-N) conditions. Nitrogen-starved cells were sampled for imaging after 12 and 24 hours. Examples of contacts between the nucleus and vacuoles as well as alterations in nuclear morphology are presented. N, nucleus; V, vacuole. Bar, 2 µm. (**D**) Wild type, *atg3*Δ and *atg4*Δ cells expressing n-Rosella were starved in SD(−N) medium for 0 and 24 hours, and the levels of free GFP degradation product monitored by immunoblotting as described in Materials and Methods. Cytosolic PGK was detected as a loading control. *indicates the presumptive degradation product of n-Rosella lacking the NAB35 nuclear targeting signal. **indicates non-specific degradation product observed only in growing cells (0 hours).

**Figure 8:**
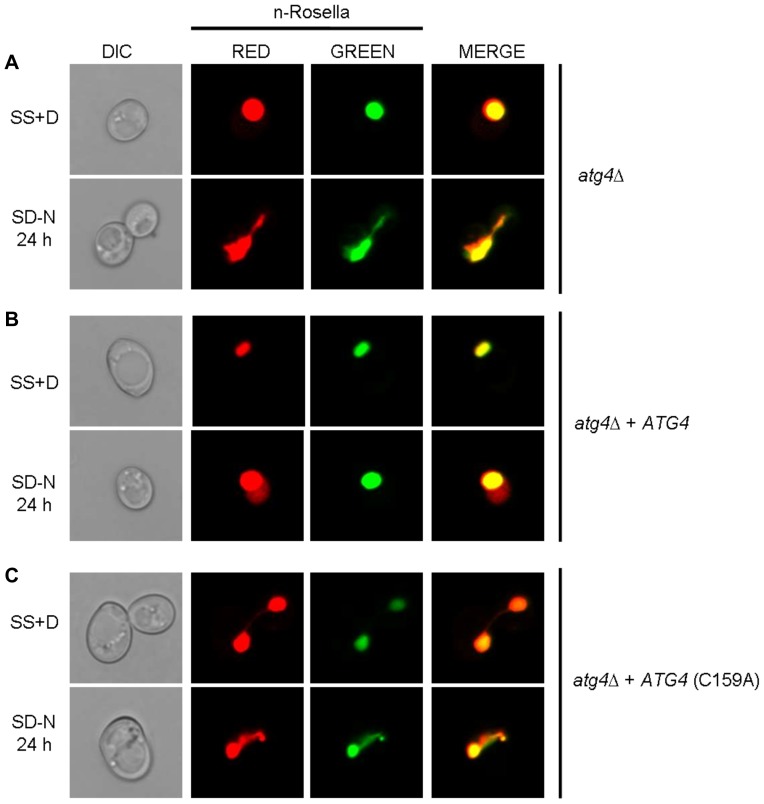
The absence of a functional *ATG4* gene product abrogates the delivery of n-Rosella to the vacuole and influences nuclear morphology. *atg4*Δ (**A**), *atg4*Δ + *ATG4* (**B**), and *atg4*Δ + Atg4C159A (**C**) cells expressing n-Rosella were imaged under growing (SS+D) and nitrogen starvation (SD−N) conditions (24 hours after commencement of nitrogen starvation).

To confirm that it is the absence of the *ATG* gene product that prevents delivery of the reporter to the vacuole, as well as affecting nuclear morphology, a plasmid-borne *ATG3* or *ATG4* gene cassette was introduced into *atg3*Δ or *atg4*Δ cells, respectively. Under conditions of nitrogen starvation, the ability of cells to deliver n-Rosella to the vacuole was restored in both instances (Fig. 7A–C and Fig. 8A–B). Diffuse red fluorescence was observed in the vacuole of nitrogen-starved cells at proportions comparable to that of starved wild type cells (∼25–35%). In addition, these ‘rescued’ cells showed normal nuclear morphology. Nuclear morphology of wild type, *atg3*Δ and *ATG3* rescue cells was also confirmed by transmission electron microscopy. Compared to the round-shaped nucleus of the wild type and *ATG3* rescue cells, the nucleus in *atg3*Δ cells sampled at 12 and 24 hours of nitrogen starvation looks extended and distorted (Fig. 7C). Furthermore, expression of enzymatically inactive variants of Atg4p; Atg4C159A (Fig. 8C) or Atg4C159S (data not shown), respectively, in *atg4*Δ cells, did not rescue the phenotype. In these cells, n-Rosella was not delivered to the vacuole under nitrogen starvation and approximately 35% of cells for each mutant exhibited altered nuclear morphology. Immunoblotting analysis of lysates prepared from *atg3*Δ or *atg4*Δ cells did not detect any free GFP even after 24 hours of nitrogen starvation. (Fig. 7D and Figs. S2B and S3D). Collectively, these results indicate that functional *ATG* gene products from Class II including *ATG3* and *ATG4* are necessary for efficient LN.

### Genes Required for ‘Classical Microautophagy’ are not Essential for LN

We investigated LN in strains lacking expression of individual components of either the VTC or EGO complex. The VTC complex is required for homotypic vacuolar fusion [30], and the EGO complex is essential for the induction of a microautophagy-like recovery process occurring after rapamycin-induced macroautophagy [13]. In each case the outcome was essentially equivalent to that found for wild type cells subjected to nitrogen starvation; accumulation of diffuse red fluorescence in the vacuole and normal nuclear morphology (Table 1). Lysates prepared from *ego1*Δ or *vtc4*Δ cells and subjected to immunoblotting analysis showed that after 24 hours of nitrogen starvation the amount of free GFP detected was similar to that observed in wild type cells (Figs. S2B and S4E). These results indicate that the components of both complexes are not required for LN during nitrogen starvation-induced autophagy.

## Discussion

Table 2 summarizes the key differences between LN and PMN [31]. It is to be emphasized that this study shows that nuclear delivery to the vacuole observed using nucleoplasm reporters (n-Rosella and NAB35-DsRed.T3) is different from PMN because there is no requirement for the NV junction components, Nvj1p or Vac8p, and does not require Vps34 PtdIns(3)P-kinase complex I components (e.g., Vps34, Vps15, Atg6) and or other core *ATG* genes, for example *ATG11*, essential for PMN. Furthermore, LN is temporally and spatially distinct from PMN. Thus, we have concluded that two forms of nucleophagy can occur independently in nitrogen-starved yeast cells.

**Table 2:** Major requirement differences between PMN and LN.

Requirement	PMN	Late Nucleophagy (LN)
Cvt-specific protein Atg11	YES	NO
*NVJ1* and *VAC8* genes	YES	NO
Phosphatidylinositol 3-kinase (PI3K) complex I	YES	NO
Altered nuclear morphology	Not reported	YES
Nuclear vesicle budding	YES	Mechanism not determined
Minimal time required for vacuolar delivery	3–6 hours	20–24 hours

### The Requirement for *ATG* Genes in LN

*ATG* genes required for effective LN belong to class II (Table 1). Our work identifies an additional role for some of the encoded Atg proteins in terms of their requirement for selective degradation of the nucleus by LN. The genes required encode components belonging to the two ubiquitin-like (Atg5p-Atg12p and Atg8p) conjugation pathways and the Atg9p cycling system. Notable members of the core autophagy gene group not required for LN is *ATG6*, which together with *VPS15* and *VPS34* (Fig. S2B) comprise PtdIns(3)P-kinase complex I (Table 1). That this complex can be dispensed for LN suggests that part of the machinery required to initiate macroautophagic events is not required.

All four components of the Atg8p-phosphatidylethanolamine (PE) conjugation system, namely *ATG3*, *ATG4*, *ATG7* and *ATG8*, are essential for efficient LN (Table 1), suggesting the generation of Atg8p-PE is important for the process by which LN takes place. Confirmation of the requirement for the enzymatic activities for some of these Atg proteins was determined by testing for LN in null cells expressing enzymatically inactive Atg protein. For example, variants of Atg4p (i.e., C159A or C159S) [18] are not able to “rescue” the *atg4*Δ phenotype. Presently it is unclear how Atg8p-PE might facilitate uptake of nucleoplasm into the vacuole, but an attractive possibility is that it acts to bring the two opposing (vacuolar and nuclear) membranes into close contact for interaction and eventual fusion. Indeed, Atg8p has been demonstrated to be involved in tethering between adjacent membranes and stimulating membrane hemifusion *in vitro*, although the physiological relevance of this activity has not been determined [32], [33]. Such functions, which mimic expansion of the autophagosomal membrane during macroautophagy, may contribute to membrane interactions during LN. What is the role of the Atg12p–Atg5p–Atg16p complex in LN? Previous suggestions made in the context of canonical macroautophagy [34] could apply to LN, namely that this complex acts as a coat component to drive curvature of membranes during LN puncta formation. Alternatively the primary importance of the Atg12p–Atg5p conjugate could be to function as an E3, ubiquitin ligase for Atg8p-PE conjugation [35].

Two components of the Atg9p cycling systems, namely Atg11p and Atg27p, are dispensable for LN whereas Atg2p, Atg18p and Atg1p and Atg13p (Table 1) are required for LN. In growing conditions the absence of Atg11p blocks transport of Atg9p to the pre-autophagosome (PAS) [36], [37]. By contrast, under starvation conditions, Atg9 recruitment to the PAS does not require Atg11, but requires interaction with Atg17 [38] which we found to be essential for LN. The anterograde movement of Atg9p to the PAS also depends on Atg23p and Atg27p [39], [40]. Atg27 is reported to shuttle between the PAS, mitochondria, and the Golgi complex. Presumably membrane from these sources is not required for LN as Atg27 was shown to be dispensable for LN.

If Atg9p localization at the PAS under starvation conditions is required for LN then the requirement for the Atg1p kinase complex, Atg18p and Atg2p may relate to these autophagy components being normally required for the retrograde movement of Atg9p away from the PAS [41]. Also one might speculate that Atg2p and Atg18p, as peripheral membrane proteins, may localize to the nuclear envelope (NE) and/or vacuolar membrane to co-operate with Atg9p and Atg8-PE to facilitate membrane interactions between vacuole and the nucleus. In this context the role of the Atg1p-Atg13p complex could be, as previously reported [42], to promote the interaction of Atg9p with Atg2p and Atg18p. It is possible that other proteins yet to be identified associate with Atg1p and/or Atg13p to facilitate the process of LN. Vac8p cannot act in such a capacity since it is not required for LN (Table 1), despite it being a putative binding partner of Atg13p.

Notably, there are proteins that are not required for LN, but which are required for PMN. The Cvt-specific protein, Atg11p is not required and hence LN is not a Cvt related process. Taken together these differences clearly distinguish LN mechanistically not only from PMN, but also from Cvt thus defining LN as a new selective, starvation-induced autophagic subtype. However, there remains much to be determined about the precise mechanism and timing of membrane re-arrangements driving autophagic turnover of the nucleus during LN, in particular the localization of those Atg proteins essential for LN and the order of their recruitment to the site of LN puncta production.

A related question is the possible role of lipid trafficking membrane proteins and their possible contribution to the mechanism of LN. In yeast Osh proteins have been proposed to play a general role in lipid trafficking at membrane contact sites between different organelles including the nucleus and vacuole [43], [44]. Goldfarb and colleagues [5] showed that upon nitrogen starvation, and concomitant with increased expression of Nvj1p, two proteins, Osh1p and Tsc13p, required for PMN [6], [45], [46] are recruited to NV junctions presumably in order to facilitate lipid biosynthesis and trafficking. Whether such proteins are required for LN has not yet been determined, however, if these proteins are recruited for LN then they must interact with a different subset of proteins given that Nvj1p is not required for LN.

### The Nature of LN Puncta

Although PMN vesicles, and presumably LN puncta, are formed from components of both the NE and vacuolar membrane, their structure appears to be different. For example, in the vacuole PMN vesicles appear more stable than LN puncta. LN derived puncta can be visualized in *atg15*Δ (Fig. 5A) and *atg22*Δ cells (Table 1), *pep4*Δ (Fig. 5D–F) cells or in wild type cells incubated with the cell permeable serine protease inhibitor, PMSF (data not shown), that inhibits the breakdown of autophagic vesicles in the vacuole. This result suggests that the membrane complement of proteins and lipids of PMN vesicles makes them more resistant to degradation (or dis-assembly). Such properties might arise from the presence of one or both of the membranes (nuclear and vacuolar derived) that presumably contribute to the structure of the vesicles/puncta. At present we can only speculate regarding any membrane surrounding LN puncta that can be observed in *atg15*Δ or *pep4*Δ cells. Potentially differences may arise because vesicle or puncta formation involves recruitment of different sub-domains of the NE and/or the vacuolar membrane. For PMN, NE regions lacking the nuclear pore complexes but containing smooth ER-derived membranes with a few integral membrane proteins were reported by Thumm and colleagues [46] as contributing to PMN vesicles.

### Alterations in Nuclear Morphology During LN

Cells unable to deliver material to the vacuole by LN (e.g., *atg3*Δ cells) show altered nuclear morphology. It is not clear whether these alterations are a consequence of LN being mechanistically deranged leading to other alternative outcomes or the inability of cell to remove material from the nucleus. Normal nuclear morphology was observed in *atg15*Δ (Fig. 5A) and/or *atg22*Δ cells (data not shown) or wild type cells treated with PMSF (data not shown) in which material is delivered to the vacuole, but not degraded. It is possible these observations reflect an excess of nuclear envelope or the presence of stress prone components of the nucleus in these cells that would normally be removed during nitrogen starvation induced autophagy.

Furthermore, the presence of protrusions or ‘horns’ evident after nitrogen starvation can be reversed if cells are taken out of nitrogen starvation and put back into SS+D medium (data not shown). This indicates that alterations in nuclear morphology arise largely as a consequence of nitrogen starvation rather than a deficiency in nucleophagy. Similar alterations in nuclear morphology to those observed here were reported in *spo7*Δ and *nem1*Δ [47]–[49]. The *SPO7* or *NEM1* genes encode proteins that are essential for the maintenance of normal spherical nuclear morphology, but it is unclear why loss of these proteins has an effect on nuclear structure.

In addition, we investigated whether altered nuclear morphology might have arisen as a consequence of yeast apoptosis-like processes occurring after extended nitrogen starvation. Propidium iodide nucleic acid stain was used to identify dead cells [50] in wild type, *atg3*Δ and *atg11*Δ cultures grown in SS+D or SD−N medium for 24 hrs. We found less than 5% dead cells in all of these cell populations (Fig. S5), whereas the percentage of cells showing LN and/or changes in nuclear morphology was much greater (25–35%). Our conclusion is that apoptosis-like processes do not contribute to altered nuclear morphology.

### Other Questions Concerning the Molecular Mechanism of LN

A significant question is whether PMN and LN facilitate the sequestration of different nuclear cargoes. At present we have no clues as to the cargo delivered to the vacuole in LN. This can be addressed by determining if different nuclear components such as histones, nucleolus and nuclear pore complexes are degraded under conditions including PMN and LN. We have established that canonical NV junctions are not required for LN, but it remains to be definitively established whether both PMN and LN may occur at the same regions of nucleus-vacuole interaction. The temporal separation observed indeed suggests that LN and PMN are largely exclusive events perhaps requiring different vacuolar/nuclear contacts and associated components (proteins and/or lipids). Future studies will be directed towards elucidating the nature of the nucleus-vacuole contact during LN, particularly in the absence of Vac8p and Nvj1p proteins, as well any mechanistic and/or functional inter-relationship of LN and PMN.

## Supporting Information

Figure S1 (Higher magnification images corresponding to Figures 3A and 3C, respectively.) (**A**) Wild type (BY4741) cells co-expressing both nuclear reporters were imaged under growing (SS+D) and nitrogen starvation (SD-N) conditions (3 and 24 hours after commencement of nitrogen starvation), respectively. The appearance of Nvj1p-EYFP-derived vesicles (PMN blebs and/or vesicles) in the vacuole is highlighted by white arrows, whereas accumulation of NAB35-DsRed.T3-derived fluorescence (diffuse red fluorescence) is indicated by yellow arrows. (**B**) Accumulation of both Nvj1p-EYFP-derived vesicles (PMN blebs and/or vesicles) and accumulation of NAB35-DsRed.T3-derived (diffuse red) fluorescence in the same cells 24 hours after commencement of nitrogen starvation. The appearance of vacuolar vesicles containing both nuclear reporters is indicated by white arrows.
(TIF)

Figure S2 Percentage of cells showing accumulation of red fluorescence in the vacuole under growing conditions and 24 hours after commencement of nitrogen starvation for wild type and *atg* null mutant strains (A), and other null mutants strains (B). (C) levels of free GFP degradation product monitored by immunoblotting as described in Materials and Methods. Cytosolic PGK was detected as a loading control. *indicates the presumptive degradation product of n-Rosella lacking the NAB35 nuclear targeting signal. **indicates non-specific degradation product observed only in growing cells (0 hours).
(TIF)

Figure S3 (**A**) *vac17*Δ, *tco89*Δ, (**B**) wild type (BY4741), *atg6*Δ, *atg11*Δ (**C**) wild type (BY4741), *atg8*Δ, *atg10*Δ and (**D**) wild type (BY4741), *atg3*Δ, *atg4*Δ cells expressing n-Rosella were starved in SD(-N) medium for 0, 6, 12, and 24 hours. The level of free GFP degradation product was monitored by immunoblotting as described in Materials and Methods. * indicates the presumptive degradation product of n-Rosella lacking the NAB35 nuclear targeting signal. Cytosolic PGK was detected as a loading control in panel A.
(TIF)

Figure S4 (Higher magnification images corresponding to the Figure 5A, 5B, 5D and 5E, respectively.) (**A**) *atg15*Δ cells expressing n-Rosella were imaged under growing (SS+D) and nitrogen starvation (SD-N) conditions (24 hours after commencement of nitrogen starvation). Staining with Hoechst 33258 was performed to confirm the targeting of n-Rosella (red and green fluorescence) to the nucleus (24 hours after commencement of nitrogen starvation) and nucleus-derived vesicles/puncta observed in the vacuole. (**B**) *atg15*Δ cells co-expressing the nuclear reporters, Nvj1-EYFP and NAB35-DsRed.T3 were imaged under growing (SS+D) and nitrogen starvation (SD-N) conditions (24 hours after commencement of nitrogen starvation). White arrow highlights Nvj1p-EYFP labeled vesicle whereas yellow arrow highlights NAB35-DsRed.T3 labeled vesicle/puncta, respectively. (**C**) *pep4*Δ cells expressing n-Rosella were imaged under growing (SS+D) and nitrogen starvation (SD-N) conditions (24 hours after commencement of nitrogen starvation). (**D**) *pep4*Δ cells co-expressing the nuclear reporters, Nvj1-EYFP and NAB35-DsRed.T3 were imaged under growing (SS+D) and nitrogen starvation (SD-N) conditions (24 hours after commencement of nitrogen starvation). The appearance of Nvj1p-EYFP-derived vesicles (PMN blebs and/or vesicles) in the vacuole is highlighted by white arrows, whereas accumulation of NAB35-DsRed.T3-derived vesicles/puncta is indicated by yellow arrows. (**E**) Wild type (BY4741), *ego1*Δ, *vtc4*Δ, *atg15*Δ and *pep4*Δ cells expressing n-Rosella were starved in SD(-N) medium for 0 and 24 hours, and the levels of free GFP degradation product monitored by immunoblotting as described in Materials and Methods. *indicates the presumptive degradation product of n-Rosella lacking the NAB35 nuclear targeting signal. Cytosolic PGK was detected as a loading control.
(TIF)

Figure S5 Wild type (BY4741), *atg3*Δ and *atg11*Δ cells were grown in SS+D or SD-N medium for 24 hours. Loss of plasma membrane integrity (indication of dead cells) was indicated by PI staining. Cells were incubated with 5 µg/ml of PI for 10 min at room temperature.
(TIF)
