# Supplementary figures and images for: A Late Form of Nucleophagy in Saccharomyces cerevisiae
Source: PLoS One. 2012 Jun 29;7(6):e40013. doi: 10.1371/journal.pone.0040013 (PMC3386919; doi:10.1371/journal.pone.0040013)

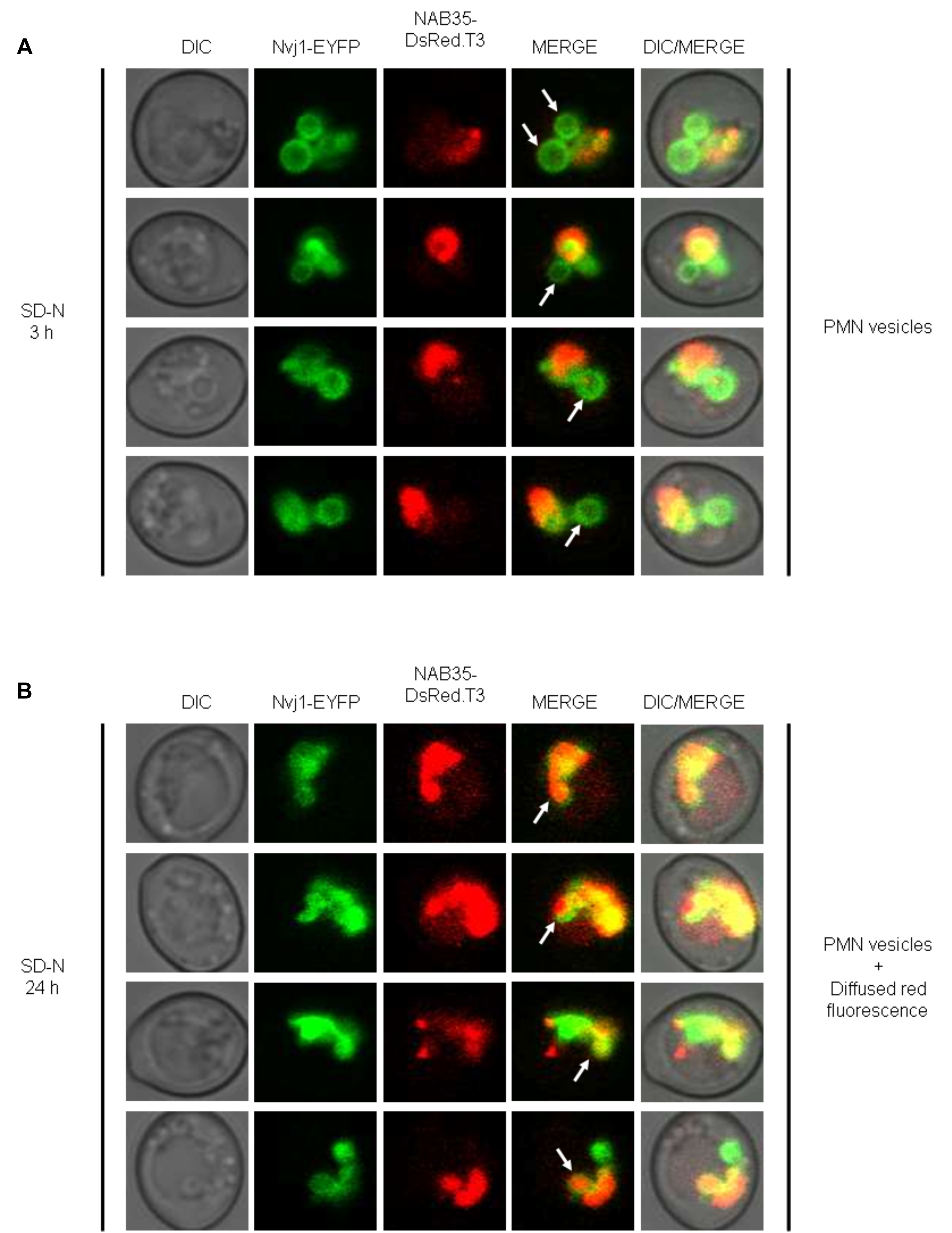

Supplement: Figure S1 — (Higher magnification images corresponding to Figures 3A and 3C, respectively.) (A) Wild type (BY4741) cells co-expressing both nuclear reporters were imaged under growing (SS+D) and nitrogen starvation (SD-N) conditions (3 and 24 hours after commencement of nitrogen starvation), respectively. The appearance of Nvj1p-EYFP-derived vesicles (PMN blebs and/or vesicles) in the vacuole is highlighted by white arrows, whereas accumulation of NAB35-DsRed.T3-derived fluorescence (diffuse red fluorescence) is indicated by yellow arrows. (B) Accumulation of both Nvj1p-EYFP-derived vesicles (PMN blebs and/or vesicles) and accumulation of NAB35-DsRed.T3-derived (diffuse red) fluorescence in the same cells 24 hours after commencement of nitrogen starvation. The appearance of vacuolar vesicles containing both nuclear reporters is indicated by white arrows. (TIF) [file pone.0040013.s001.tif]

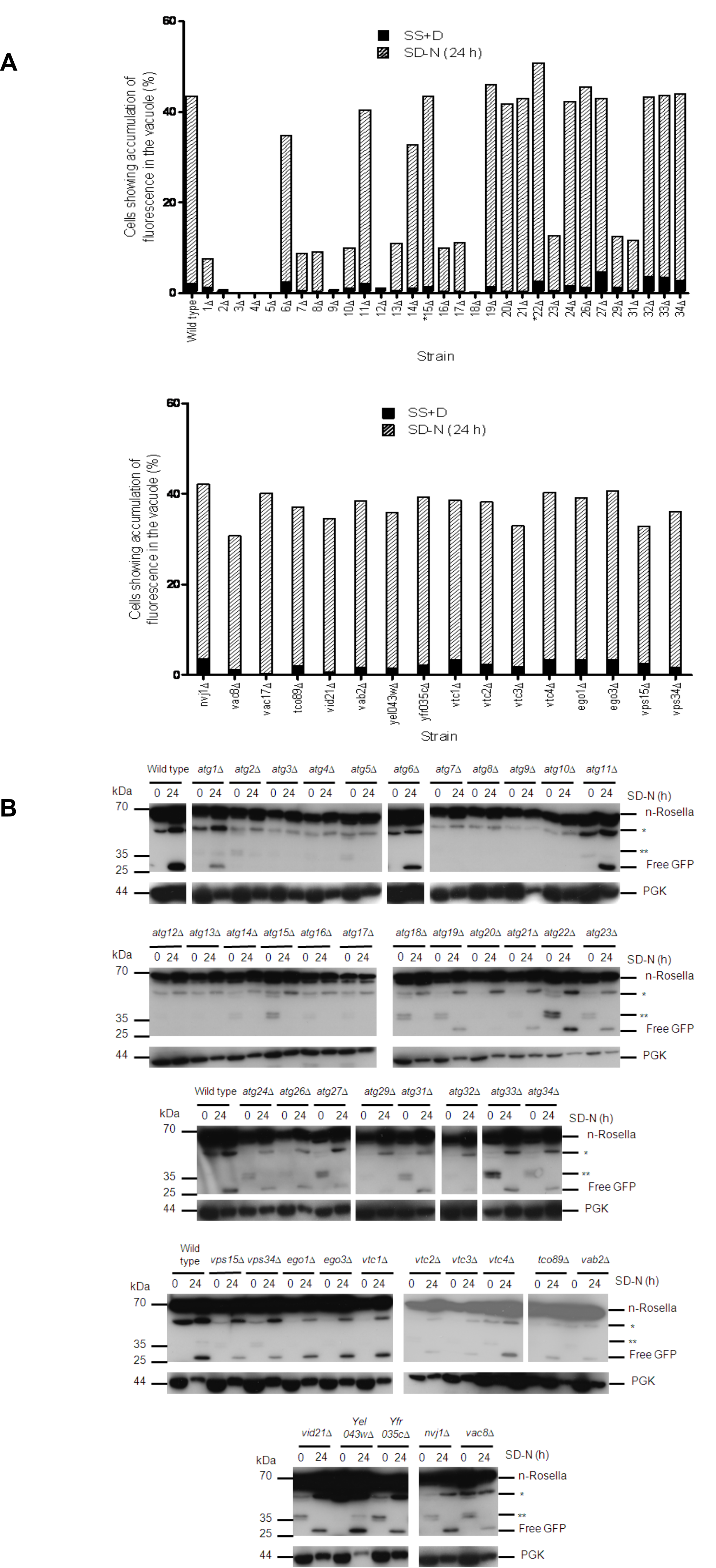

Supplement: Figure S2 — Percentage of cells showing accumulation of red fluorescence in the vacuole under growing conditions and 24 hours after commencement of nitrogen starvation for wild type and atg null mutant strains (A), and other null mutants strains (B). (C) levels of free GFP degradation product monitored by immunoblotting as described in Materials and Methods. Cytosolic PGK was detected as a loading control. *indicates the presumptive degradation product of n-Rosella lacking the NAB35 nuclear targeting signal. **indicates non-specific degradation product observed only in growing cells (0 hours). (TIF) [file pone.0040013.s002.tif]

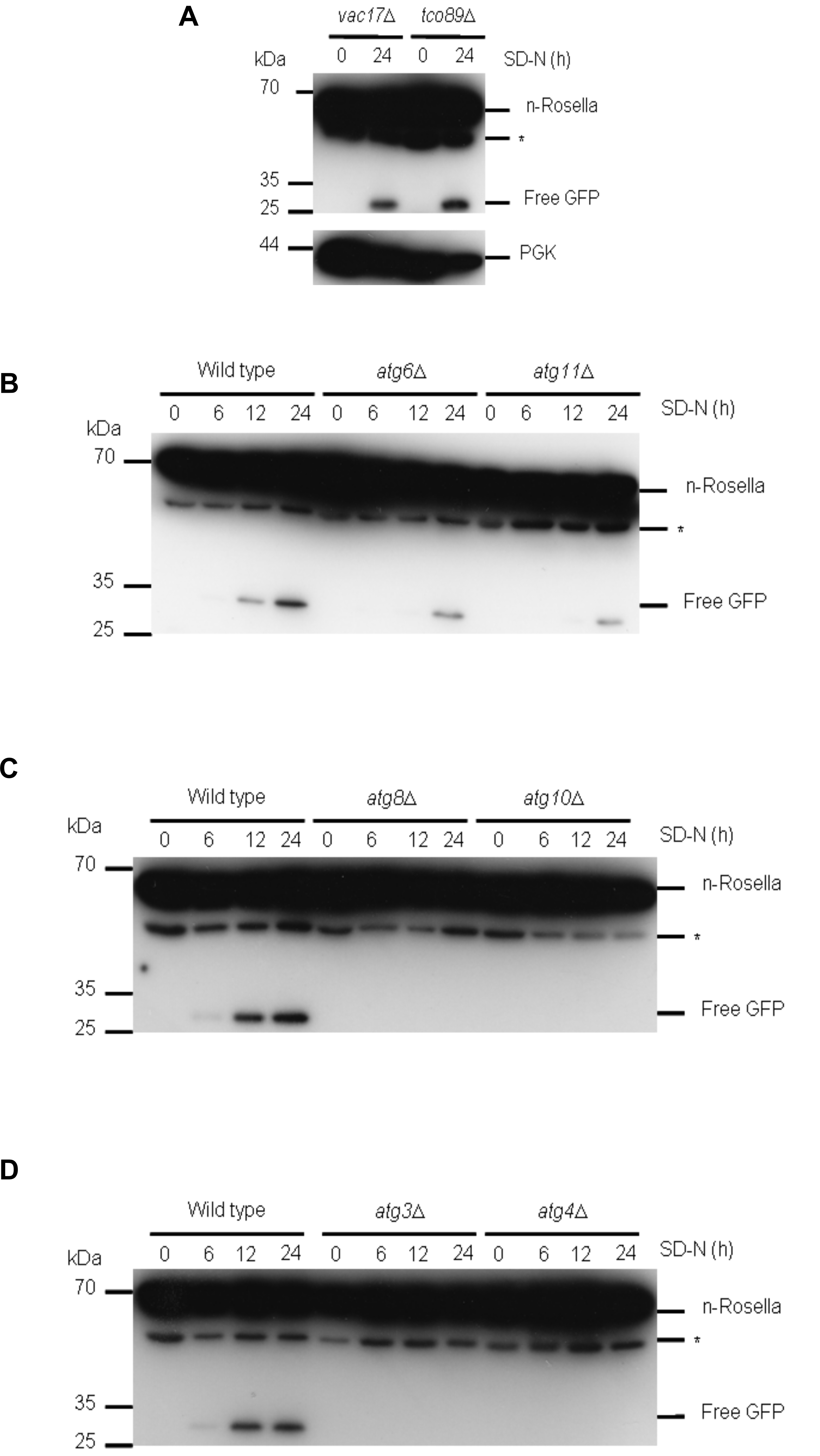

Supplement: Figure S3 — (A) vac17Δ, tco89Δ, (B) wild type (BY4741), atg6Δ, atg11Δ (C) wild type (BY4741), atg8Δ, atg10Δ and (D) wild type (BY4741), atg3Δ, atg4Δ cells expressing n-Rosella were starved in SD(-N) medium for 0, 6, 12, and 24 hours. The level of free GFP degradation product was monitored by immunoblotting as described in Materials and Methods. * indicates the presumptive degradation product of n-Rosella lacking the NAB35 nuclear targeting signal. Cytosolic PGK was detected as a loading control in panel A. (TIF) [file pone.0040013.s003.tif]

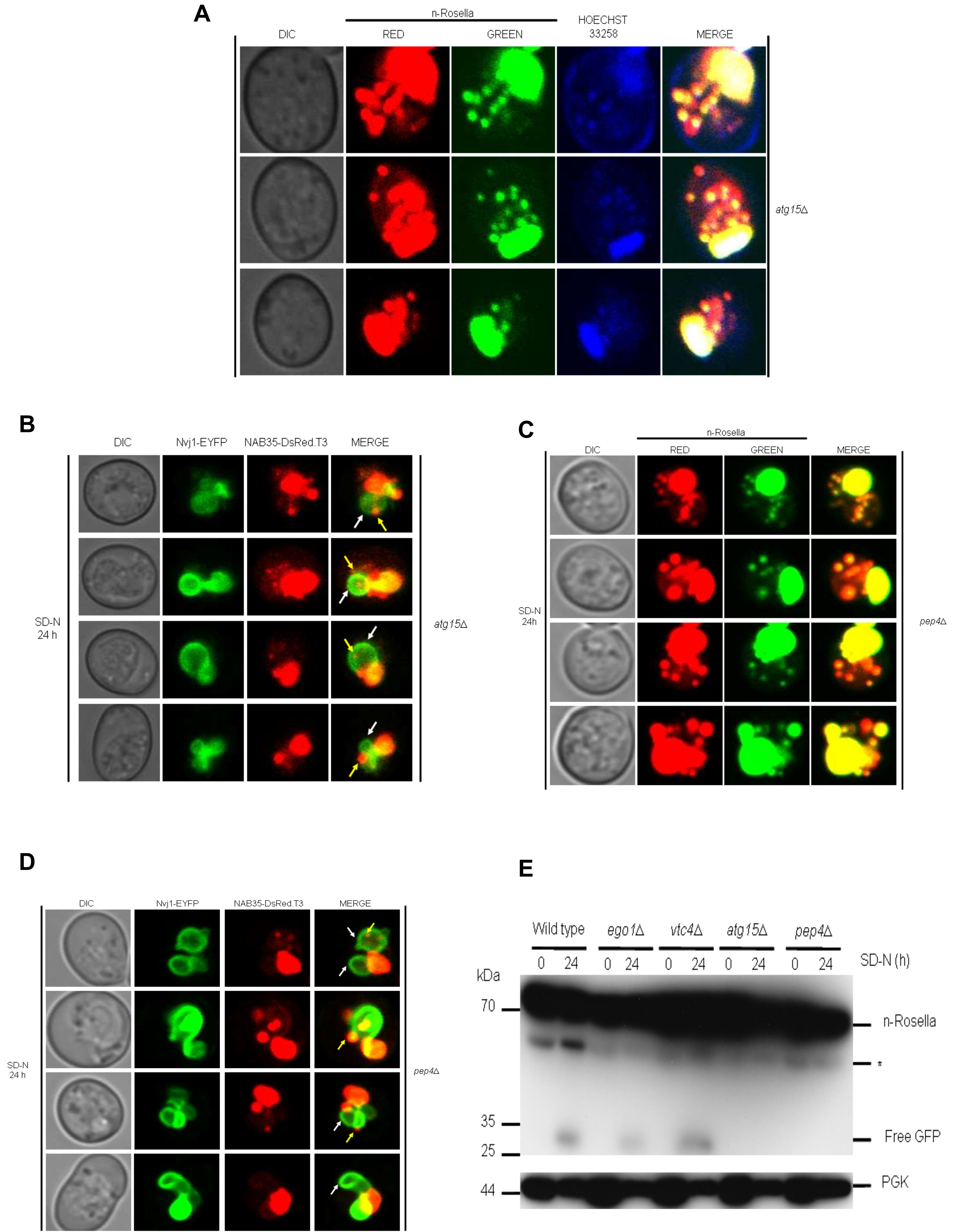

Supplement: Figure S4 — (Higher magnification images corresponding to the Figure 5A, 5B, 5D and 5E, respectively.) (A) atg15Δ cells expressing n-Rosella were imaged under growing (SS+D) and nitrogen starvation (SD-N) conditions (24 hours after commencement of nitrogen starvation). Staining with Hoechst 33258 was performed to confirm the targeting of n-Rosella (red and green fluorescence) to the nucleus (24 hours after commencement of nitrogen starvation) and nucleus-derived vesicles/puncta observed in the vacuole. (B) atg15Δ cells co-expressing the nuclear reporters, Nvj1-EYFP and NAB35-DsRed.T3 were imaged under growing (SS+D) and nitrogen starvation (SD-N) conditions (24 hours after commencement of nitrogen starvation). White arrow highlights Nvj1p-EYFP labeled vesicle whereas yellow arrow highlights NAB35-DsRed.T3 labeled vesicle/puncta, respectively. (C) pep4Δ cells expressing n-Rosella were imaged under growing (SS+D) and nitrogen starvation (SD-N) conditions (24 hours after commencement of nitrogen starvation). (D) pep4Δ cells co-expressing the nuclear reporters, Nvj1-EYFP and NAB35-DsRed.T3 were imaged under growing (SS+D) and nitrogen starvation (SD-N) conditions (24 hours after commencement of nitrogen starvation). The appearance of Nvj1p-EYFP-derived vesicles (PMN blebs and/or vesicles) in the vacuole is highlighted by white arrows, whereas accumulation of NAB35-DsRed.T3-derived vesicles/puncta is indicated by yellow arrows. (E) Wild type (BY4741), ego1Δ, vtc4Δ, atg15Δ and pep4Δ cells expressing n-Rosella were starved in SD(-N) medium for 0 and 24 hours, and the levels of free GFP degradation product monitored by immunoblotting as described in Materials and Methods. *indicates the presumptive degradation product of n-Rosella lacking the NAB35 nuclear targeting signal. Cytosolic PGK was detected as a loading control. (TIF) [file pone.0040013.s004.tif]

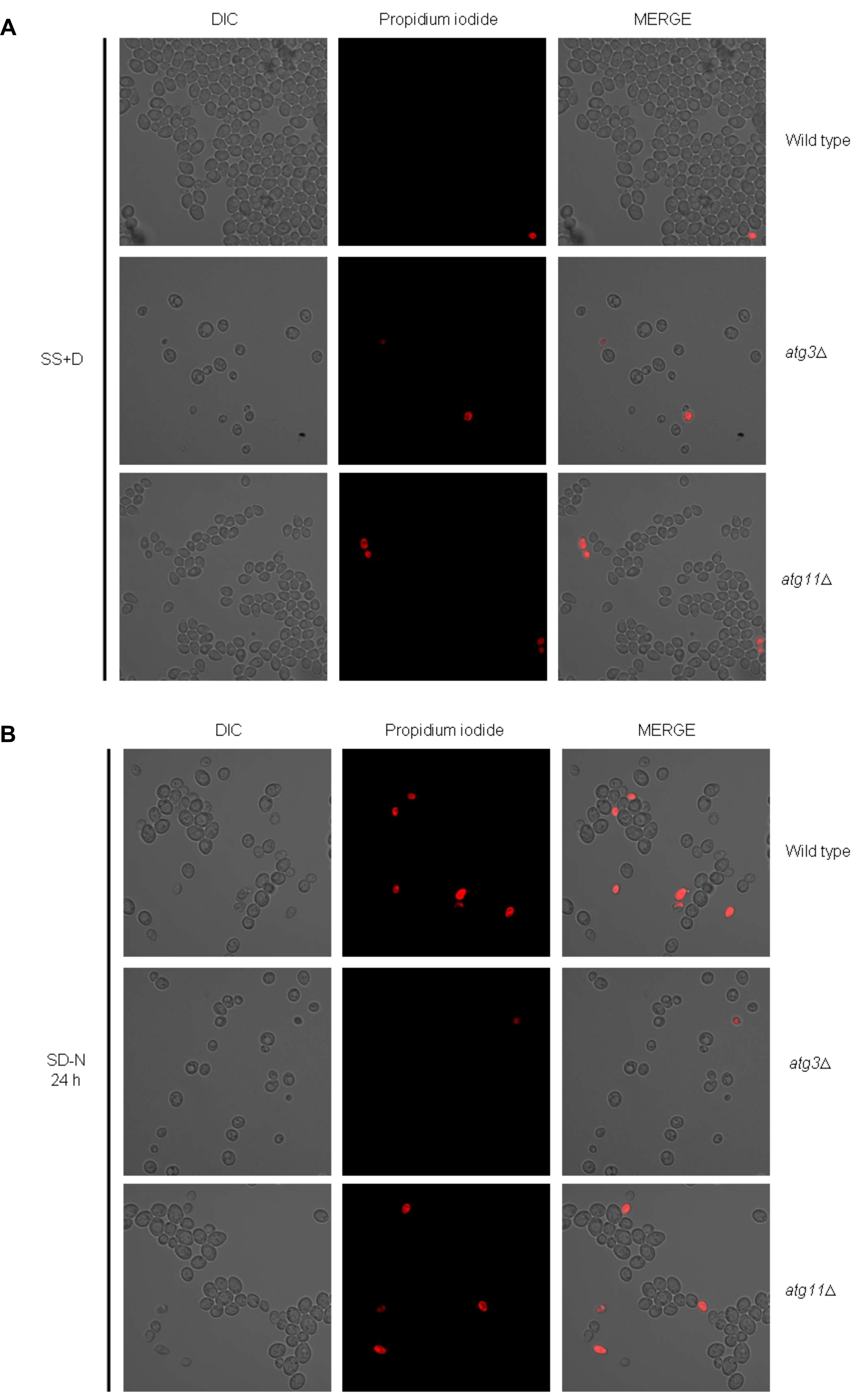

Supplement: Figure S5 — Wild type (BY4741), atg3Δ and atg11Δ cells were grown in SS+D or SD-N medium for 24 hours. Loss of plasma membrane integrity (indication of dead cells) was indicated by PI staining. Cells were incubated with 5 µg/ml of PI for 10 min at room temperature. (TIF) [file pone.0040013.s005.tif]
